# Creation of Machine Learning Models Trained on Multimodal Physiological, Behavioural, Blood Biochemical, and Milk Composition Parameters for the Identification of Lameness in Dairy Cows

**DOI:** 10.3390/bios15110722

**Published:** 2025-10-31

**Authors:** Karina Džermeikaitė, Justina Krištolaitytė, Samanta Grigė, Akvilė Girdauskaitė, Greta Šertvytytė, Gabija Lembovičiūtė, Mindaugas Televičius, Vita Riškevičienė, Ramūnas Antanaitis

**Affiliations:** 1 Animal Clinic, Veterinary Academy, Lithuania University of Health Sciences, Tilžės Str. 18, LT-47181 Kaunas, Lithuania; justina.kristolaityte@lsmu.lt (J.K.); samanta.grige1@lsmu.lt (S.G.); akvile.girdauskaite@lsmu.lt (A.G.); greta.sertvytyte@lsmu.lt (G.Š.); gabija.lemboviciute@lsmu.lt (G.L.); mindaugas.televicius@lsmu.lt (M.T.); ramunas.antanaitis@lsmu.lt (R.A.); 2 Department of Veterinary Pathobiology, Faculty of Veterinary, Veterinary Academy, Lithuanian University of Health Sciences, Tilžės Str. 18, 44307 Kaunas, Lithuania; vita.riskeviciene@lsmu.lt

**Keywords:** artificial intelligence, biosensors, blood biomarkers, milk quality, precision livestock farming

## Abstract

Lameness remains a significant welfare and productivity challenge in dairy farming, often underdiagnosed due to the limitations of conventional detection methods. Unlike most previous approaches to lameness detection that rely on a single-sensor or gait-based measurement, this study integrates four complementary data domains—behavioural, physiological, biochemical, and milk composition parameters—collected from 272 dairy cows during early lactation to enhance diagnostic accuracy and biological interpretability. The main objective of this study was to evaluate and compare the diagnostic classification performance of multiple machine learning (ML) algorithms trained on multimodal data collected at the time of clinical lameness diagnosis during early lactation, and to identify the most influential physiological and biochemical traits contributing to classification accuracy. Specifically, six algorithms—random forest (RF), neural network (NN), Ensemble, support vector machine (SVM), k-nearest neighbors (KNN), and logistic regression (LR)—were assessed. The input dataset integrated physiological parameters (e.g., water intake, body temperature), behavioural indicators (rumination time, activity), blood biochemical biomarkers (non-esterified fatty acids (NEFA), aspartate aminotransferase (AST), lactate dehydrogenase (LDH), gamma-glutamyl transferase (GGT)), and milk quality traits (fat, protein, lactose, temperature). Among all models, RF achieved the highest validation accuracy (97.04%), perfect validation specificity (100%), and the highest normalized Matthews correlation coefficient (nMCC = 0.94), as determined through Monte Carlo cross-validation on independent validation sets. Lame cows showed significantly elevated NEFA and body temperatures, reflecting enhanced lipid mobilization and inflammatory stress, alongside reduced water intake, milk protein, and lactose content, indicative of systemic energy imbalance and impaired mammary function. These physiological and biochemical deviations emphasize the multifactorial nature of lameness. Linear models like LR underperformed, likely due to their inability to capture the non-linear and interactive relationships among physiological, biochemical, and milk composition features, which were better represented by tree-based and neural models. Overall, the study demonstrates that combining sensor data with blood biomarkers and milk traits using advanced ML models provides a powerful, objective tool for the clinical classification of lameness, offering practical applications for precision livestock management by supporting early, data-driven decision-making to improve welfare and productivity on dairy farms.

## 1. Introduction

Lameness is one of the most prevalent and economically significant health issues affecting dairy farms worldwide. It compromises not only animal welfare but also reproductive efficiency, milk production, and the overall sustainability of dairy operations [1]. Despite its major impact, lameness often remains underdiagnosed, especially during its early stages, primarily due to the limitations of conventional detection methods. Visual locomotion scoring—the most commonly used approach—is subjective, labour-intensive, and highly dependent on the observer’s experience, leading to inconsistent and delayed identification of affected animals [2,3]. It has been shown that 55% of lactations are linked to lameness-related health issues, and 15% to mastitis or uterine infections. This indicates that, despite extensive information regarding dairy cow management, some unresolved challenges persist [4]. Biochemical markers such as non-esterified fatty acids (NEFA) and beta-hydroxybutyrate (BHB) reflect energy balance and are linked to postpartum metabolic disorders, which can affect cow health, productivity, and welfare [5]. Lameness not only affects locomotion but also notably alters cow behaviour—lame dairy cows spend less time feeding, exhibit decreased feed intake, and are less active overall, while rumination time is significantly reduced [5,6]. These behavioural changes often correspond with declines in milk yield, and milk composition is also altered, with lame cows producing less milk and showing higher fat and lower lactose content compared to healthy cows [6].

In recent years, the dairy industry has undergone a technological transformation, with increasing adoption of precision livestock farming (PLF) tools that generate high-frequency, individualized health and productivity data [7]. This advancement opens new opportunities to develop objective, data-driven diagnostic solutions. Yet, most existing efforts to apply data analytics to lameness detection have focused on single domains—such as motion sensors, video-based gait analysis, or milk yield deviations—providing only a partial understanding of the complex physiological disruptions associated with lameness [8,9]. Few studies have attempted to combine diverse biological and production-related indicators into a unified, interpretable model for classifying clinically lame versus healthy cows.

Machine learning (ML), a subfield of artificial intelligence, employs statistical methods to identify or forecast bovine performance or illness occurrences by utilising extensive datasets and managing complex relationships arising from an ever-expanding array of variables [10]. Researchers have employed ML techniques to identify or forecast many health conditions, including clinical mastitis, utilising random forest (RF), naïve Bayes, eXtreme Gradient boosting [11], neural networks (NN) [12], decision-tree induction [13], and logistic generalised linear mixed models [14]. Furthermore, the identification of metritis [15] and the assessment of metabolic status [16] in dairy cows during early lactation have been concurrently executed using five and eight ML algorithms, respectively.

The present study addresses this gap by evaluating the diagnostic performance of six ML models—RF, support vector machine (SVM), logistic regression (LR), NN, k-nearest neighbors (KNN), and an Ensemble approach—in classifying lameness based on a comprehensive, multimodal dataset. Data were collected at the time of clinical diagnosis and included physiological metrics (such as water intake, reticulorumen temperature), behavioural patterns (rumination time, cow activity), blood biomarkers (including NEFA, aspartate aminotransferase (AST), lactate dehydrogenase (LDH), and gamma-glutamyl transferase (GGT), triglycerides (TRIG), total protein (TP), lactate dehydrogenase (LDH), iron (Fe)), and milk quality parameters (such as milk protein, lactose, temperature and fat-to-protein ratio). By integrating these diverse features, this study moves beyond reductionist, single sensor approaches and reflects the multifactorial nature of lameness as a clinical condition. In ML, it is customary to assess multiple algorithms on integrated data (e.g., management, health, milking) as the efficacy of each algorithm may be influenced by features, sample size, structure, and other attributes of the data set [16]. Prior research has utilised accelerometer-derived data to predict lameness in dairy cows, revealing a minor to moderate correlation between these behavioural metrics (laying and standing) and lameness detection [17], with an accuracy of 87% [18], sensitivity of 90.2%, and specificity of 91.7% [19]. What sets this study apart is its methodological rigor and biological breadth. The integration of phenotypic and biochemical information allows for a robust evaluation of model performance, highlighting the superiority of tree-based and neural network models in capturing complex patterns associated with lameness.

The novelty of this work lies in its integrative approach, the comparative evaluation of multiple ML algorithms, and the inclusion of underexplored health indicators such as metabolic and inflammatory biomarkers. By bridging sensor data with blood chemistry and milk traits, this study introduces a new paradigm for understanding and diagnosing lameness in dairy cattle. It represents a meaningful advancement in both scientific methodology and real-world applicability, aligning with the broader goals of sustainable and welfare-oriented livestock farming.

The aim of this study was to evaluate and compare the diagnostic classification performance of six ML models—RF, SVM, LR, NN, KNN, and an Ensemble model—in distinguishing clinically lame versus healthy dairy cows during early lactation. All multimodal data, including physiological sensor measurements, behavioural traits, blood biomarkers, and milk quality parameters, were collected at the time of clinical lameness diagnosis. This design enables an objective evaluation of diagnostic model performance, rather than pre-clinical prediction, while emphasizing the biological interpretability of integrated multimodal features.

## 2. Materials and Methods

### 2.1. Ethical Approval

All animal-related procedures received approval from the Institutional Animal Care and Use Committee of the Lithuanian University of Health Sciences (LSMU) (Protocol No. G2-227, adopted on 7 March 2025). The research complied with the stipulations of EU Directive 2010/63/EU regarding the protection of animals utilised for scientific purposes.

### 2.2. Study Design and Animals

This observational case–control study was conducted at a dairy farm of Practical Training and Research Center of LSMU, using high-producing Holstein-Friesian cows in early lactation. The 5 January 2025 marked the beginning of the experiment, which lasted until the 31 August 2025. The study population consisted of two groups: cows clinically diagnosed with lameness (n = 110) and clinically healthy controls (n = 162), matched by parity and lactation stage. All cows were housed in free-stall barns under uniform management and feeding conditions. The trial period coincided with the early lactation phase (up to 60 days in milk). DeLaval milking robots, manufactured by DeLaval Inc. in Tumba, Sweden, were used for milking the cows. A mean body weight of 550 ± 45 kilograms was recorded for the cows. They were housed in stables that were made of free-stalls and had a DeLaval ventilation system (DeLaval Inc., Tumba, Sweden). In the year 2024, the average amount of milk produced by each cow was 10 310 kg, with 4.2% fat and 3.5% protein. Throughout the course of the year, the animals were fed a total mixed ration (TMR), which was designed to meet the physiological requirements that they had. Calculations were executed utilising existing formulas [20]. The feed was carefully designed by a nutritionist to guarantee that the cows had all vital elements required for optimal health and milk output. Residual feed was eliminated daily between 5 a.m. and 2 p.m. Feeding took place daily at 8:00 and 16:00. All cows had unrestricted access to water for drinking. The TMR composition is broken out in detail in Table 1 and Table 2.

The study followed a case–control design, in which clinically lame cows (cases) were identified at the time of diagnosis through routine locomotion scoring and clinical examination. Each case was matched with a healthy control cow from the same herd, at a similar stage of lactation and parity, to minimize confounding effects related to production level or physiological stage. Matching was performed within ±5 days of lactation stage. Data collection for both groups was carried out simultaneously, ensuring that environmental and management conditions were equivalent. This design allowed for a direct comparison of physiological, behavioural, biochemical, and milk quality parameters between clinically lame and healthy animals under similar herd conditions.

### 2.3. Clinical Lameness Assessment

Cows on the farm were systematically assessed for lameness, and those displaying signs of lameness were transferred to an examination pen for the study of gait-related disorders. All cows on the farm receive corrective claw clipping at least twice a year. The herd analysed in this study exhibited a history of lameness associated with foot rot, white line disease, and digital dermatitis.

The classification of cows as either healthy or lame was based on farm herd health records, which ensured that cases of lameness were reported in a timely manner. Rapid detection and notification enabled prompt intervention and treatment, thereby contributing to better herd health outcomes. Veterinary expertise was essential in confirming lameness diagnoses and delivering appropriate therapy, underlining the value of close cooperation between veterinarians and farm personnel. Although the exact interval between the first signs of lameness and veterinary examination was not precisely established, the farm was visited by a veterinarian on a daily basis, excluding weekends. This routine presence likely minimized any delay between the onset of clinical symptoms and initiation of treatment. Continuous veterinary involvement allowed for swift management of lameness cases and helped safeguard animal welfare and herd productivity. All veterinary services were provided by the Large Animal Clinic of the LSMU, where clinicians were trained to apply standardized protocols for diagnosis and therapy. Such collaboration between academic veterinary services and farm staff promoted consistent herd health management and supported more effective livestock production practices.

Lameness was evaluated using a five-point locomotion scoring system, as described by Thomsen et al. [21] and Sprecher et al. [22]. A score of 1 represented normal gait, where cows walked with even, steady strides and showed no signs of discomfort or weight shifting. A score of 2 indicated a slightly irregular gait, with subtle deviations such as shortened steps or minor inconsistencies in movement, although obvious lameness was not apparent. A score of 3 reflected mild lameness, characterized by a clearly uneven gait and visibly shortened strides in one or more limbs, suggesting moderate discomfort though the animal was still able to walk. A score of 4 corresponded to moderate lameness, where cows showed pronounced difficulty in locomotion, often reducing weight-bearing on the affected limb(s), with restricted mobility and obvious pain. Finally, a score of 5 denoted severe lameness, marked by extreme locomotor impairment, avoidance of weight-bearing, and movement achieved only with great effort, indicating substantial pain and compromised functionality.

Cows were classified according to their locomotion scores, with animals scoring 1 or 2 considered non-lame, while those with scores of 3 to 5 were categorized as lame, following the classification system proposed by Winckler and Willen [23]. Veterinary examination further specified the clinical causes of lameness, which included foot ulcers, digital dermatitis, and white line disease. Management of affected cows consisted of hoof trimming combined with pharmacological treatment [24]. Depending on the case, therapy involved either a single subcutaneous injection of ceftiofur sodium (Naxcel^®^ 200 mg/mL, Zoetis Belgium SA, Zaventem, Belgium) at a dosage of 1 mL per 30 kg body weight, or the administration of nonsteroidal anti-inflammatory drugs to relieve pain. In one case, meloxicam (Melovem^®^ 20 mg/mL, Dopharma B.V., Raamsdonksveer, The Netherlands) was administered subcutaneously at a dose of 2.5 mL per 100 kg body weight.

Altogether, 272 cows were enrolled and divided into two study groups based on health status: 162 non-lame cows and 110 lame cows. Health status was determined through veterinary assessment and the locomotion scoring system described above.

### 2.4. Data Collection and Sensor Measurements

Daily data were collected using automated PLF technologies, including the Brolis HerdLine in-line milk analyser (Brolis Sensor Technology, Vilnius, Lithuania), intraruminal SmaXtec boluses (SmaXtec Animal Care GmbH, Graz, Austria), DeLaval milking robots (DeLaval Inc., Tumba, Sweden), and the SmaXtec climate station. These systems continuously recorded milk composition, behavioural, physiological, reticulorumen, and environmental parameters (including temperature–humidity index (THI)). In addition, blood samples were collected and analysed for biochemical and inflammatory biomarkers. An overview of all collected variables, measurement methods, and instruments is presented in Table 3.

### 2.5. Blood Sampling and Biochemical Analysis

Blood samples were collected from the coccygeal vein on the day of clinical lameness diagnosis (or the corresponding day for control cows), approximately two hours after feeding. Sampling was performed during the clinical examination, with cows restrained in headlocks, and blood drawn using a needle syringe. For biochemical profiling, samples were collected into evacuated tubes without anticoagulant (BD Vacutainer^®^, Eysin, Switzerland). Samples were maintained in an upright position and permitted to coagulate at ambient temperature (~22 °C) for approximately 30 min. Blood samples were transported at +4 °C within one hour of collection to the Laboratory of Clinical Tests at the Large Animal Clinic, Veterinary Academy, LSMU, for further analysis. During the laboratory procedure, the samples were subjected to centrifugation at a force of 1500× *g* for a duration of 15 min. Subsequently, the serum was analysed by means of conventional veterinary biochemistry analysers. Randox clinical chemistry reagent kits were employed alongside an automated wet chemistry analyser (RX Daytona, Randox Laboratories Ltd., London, UK). The subsequent biomarkers were assessed: liver enzymes (AST, GGT, LDH), metabolic indicators (NEFA, TP, TRIG), and Fe. All analyses were conducted in duplicate to guarantee quality assurance.

### 2.6. Sensor-Based Monitoring of Cow and Environmental Parameters

Reticulorumen monitoring was carried out using orally administered SmaXtec boluses, which were introduced into the reticulorumen with a specialized applicator device in accordance with the manufacturer’s instructions. Prior to use, each bolus was activated, calibrated, and digitally linked to the corresponding animal’s identification number to ensure accurate data assignment. Once applied, the devices were connected to a base station that enabled uninterrupted data transmission throughout the study period.

The boluses continuously measured key physiological and behavioural parameters, including water intake, internal body temperature, rumination time, and cow activity. Data were automatically collected at ten-minute intervals, providing a detailed and dynamic profile of animal health and behaviour.

Environmental monitoring was performed simultaneously using a SmaXtec climate sensor (SmaXtec Animal Care GmbH, Graz, Austria), which continuously recorded ambient temperature and relative humidity. These values were used to calculate the THI, a widely recognized indicator of heat stress in dairy cattle. All physiological, behavioural, and environmental data were processed and integrated through the SmaXtec Messenger^®^ software (version 3.2, SmaXtec Animal Care GmbH, Graz, Austria), creating a comprehensive and real-time overview of health status and environmental conditions.

### 2.7. Milk Quality Assessment

In this investigation, the Brolis HerdLine in-line milk analyser, which was manufactured by Brolis Sensor Technology and located in Vilnius, Lithuania, was utilised to record the composition of milk. During each milking, samples of milk were taken and tested for the following parameters: protein, fat, lactose, fat-to-protein ratio, and milk temperature.

This analyser makes use of a specialised external cavity laser spectrometer that is dependent on GaSb. This spectrometer runs in the spectral range of 2100–2400 nm and may be adjusted to a wide range of wavelengths. From the beginning of the milking process until the end, it monitored the flow of milk in transmission mode continually. The chemical absorption spectra that were collected were analysed to ascertain the amounts of significant milk ingredients such as lactose, protein, and fat. This compact “mini spectroscope” can be integrated into milking stalls or robotic milking systems, hence eliminating the need for supplementary chemicals or maintenance. During each milking procedure, the milk’s composition was assessed at five-second intervals. To derive the final results reflective of the full milking session, weighted averages of fat, protein, and lactose were calculated. These values were obtained from the dynamics of milk flow.

During the calibration process, the accuracy of every Brolis HerdLine in-line milk analyser was evaluated and evaluated in the laboratory of Eurofins. When it came to fat, protein, and lactose, the root mean square error of prediction (RMSEP) values were 0.21%, 0.19%, and 0.19%, respectively.

Milk yield was automatically recorded at each milking using a DeLaval milking robot.

### 2.8. Data Processing and Feature Selection

Prior to model development, all data underwent systematic preprocessing. The dataset was first examined for missing values. Observations with more than 10% missing data were excluded, whereas sporadic missing entries (<5% of the dataset) were imputed using variable means to preserve the sample size.

To reduce multicollinearity, Pearson correlation coefficients were computed between all continuous variables. Pairs with a correlation coefficient of r > 0.90 were considered collinear, and one variable from each pair was removed based on biological relevance, measurement reliability, and interpretability.

All continuous predictor variables were standardized using z-score normalization (mean = 0, standard deviation = 1) to ensure equal weighting and improve the performance of algorithms sensitive to feature scale (e.g., SVM, KNN, NN). Categorical variables (lame vs. healthy) were binary encoded (0 = healthy, 1 = lame).

Descriptive statistics and group comparisons were performed using independent sample *t*-tests, with the significance level set at *p* < 0.05.

### 2.9. Description of ML Models and Performance Evaluation

Datasets were initially organized using Microsoft Excel and subsequently imported into Python 3.10 for statistical analysis and ML modelling. Data processing and model development were carried out using the scikit-learn library (v1.2.2) and complementary Python packages (NumPy, Pandas, Matplotlib, and Seaborn).

To evaluate model performance, the full dataset was randomly partitioned into 80% training and 20% validation sets. Stratified sampling was applied to preserve the original class distribution (healthy: 162; lame: 110) with both subsets, ensuring balanced representation of both classes in each training and validation sample. This approach minimizes the risk of bias caused by unequal class proportions and ensures fair comparison across models. Additionally, it was enforced that each validation sample contained at least one lame cow, ensuring that classification metrics could be computed reliably in every iteration.

Model performance was assessed using Monte Carlo cross-validation. Specifically, the random splitting procedure was repeated 10 times, each time generating independent training and validation sets. For each repetition, models were retrained from scratch on the new training data and evaluated on the corresponding validation set. This approach provided a more robust estimate of model generalization performance compared to a single split, as it captures variability associated with data partitioning.

For each model and repetition, performance metrics were computed on the validation set (see Section 2.9 for details). The mean and standard deviation of all evaluation metrics—accuracy, sensitivity, specificity, precision, F1 score, receiver operating characteristic–area under the curve (ROC–AUC) and Matthews correlation coefficient (MCC)—were calculated across the 10 Monte Carlo iterations to summarize the stability and overall performance of each model.

#### 2.9.1. Random Forest

RF, a tree-based ensemble learning method, was chosen due to its robustness to noisy features, ability to handle nonlinear relationships, and built-in feature importance estimation.

The model was trained with 500 decision trees (n_estimators = 500) and a maximum tree depth of 10, which provided a balance between model complexity and generalization. The Gini impurity criterion was used to evaluate split quality, and bootstrap sampling was enabled to introduce variability among trees.

Hyperparameters (n_estimators, max_depth, min_samples_split, and min_samples_leaf) were optimized using grid search during cross-validation. Feature importance rankings were later extracted to interpret which physiological, biochemical, or milk-related traits most influenced classification.

#### 2.9.2. Support Vector Machine

SVM was selected for its effectiveness in high-dimensional spaces and its ability to find non-linear decision boundaries through kernel functions. A Radial Basis Function (RBF) kernel was applied, enabling the model to capture complex patterns in the data.

Two key hyperparameters were optimized:

C (regularization parameter): controlling the trade-off between misclassification and margin width.

γ (gamma): defining the influence of single training points on decision boundaries.

A grid search explored combinations of C and γ values within a logarithmic scale (e.g., 10^−3^ to 10^3^). Standardized input features ensured appropriate scaling for kernel-based distance computations.

#### 2.9.3. Logistic Regression

LR served as a baseline linear model, enabling comparison with more complex, non-linear algorithms. An L2 regularization penalty was applied to reduce overfitting, and the ‘lbfgs’ solver was used for parameter estimation. Regularization strength (C) was optimized through grid search. Despite its simplicity, LR provides interpretable coefficients, useful for understanding the direction and magnitude of effects of individual features.

#### 2.9.4. Neural Network

A feed-forward fully connected neural network was implemented to model potentially complex, non-linear interactions between features. The network architecture consisted of:

Input layer with dimension equal to the number of features,

Two hidden layers with 64 and 32 neurons, respectively,

Output layer with one neuron using a sigmoid activation function for binary classification.

The ReLU activation function was used in hidden layers to improve gradient propagation and computational efficiency. The model was trained using the Adam optimizer with a learning rate of 0.001, binary cross-entropy loss, and a batch size of 32.

Training was performed over 200 epochs, with early stopping based on validation loss to avoid overfitting. Hyperparameters (number of layers, neurons, learning rate, batch size) were optimized through grid search combined with 5-fold cross-validation.

#### 2.9.5. k-Nearest Neighbors

KNN was included as a non-parametric, instance-based learning method that classifies new observations based on similarity to the k nearest neighbors in the feature space. The Euclidean distance metric was used to compute similarity, and distance-based weighting was applied so that closer neighbors had a greater influence on classification decisions.

The number of neighbors (k) was optimized within the range k = 3–15, and the optimal value k = 5 was selected based on cross-validation accuracy. Because KNN performance depends strongly on feature scaling, standardized z-score normalized inputs were used.

#### 2.9.6. Ensemble Model (Stacked RF + NN + SVM)

To combine the strengths of individual classifiers, an ensemble model was constructed using hard voting. The ensemble integrated the predictions from RF, SVM, NN, KNN, and LR. Each model contributed one vote, and the final classification was based on majority voting. Ensemble methods are known to improve generalization by reducing the variance of individual models, especially when combining algorithms with different inductive biases.

#### 2.9.7. Model Evaluation

Model performance was evaluated on the independent test set using the following metrics: accuracy, sensitivity (recall), specificity, precision, F1 score, ROC-AUC, and the MCC.

Confusion matrices were generated for each model to visualize the distribution of true positives, true negatives, false positives, and false negatives. The MCC was included to provide a balanced evaluation even in the presence of potential class imbalance.

All results were averaged across 10 Monte Carlo repetitions with random train–test splits to ensure the stability and reproducibility of model performance estimates. This repeated resampling approach provides a robust estimate of model generalization performance while mitigating overfitting to specific data partitions. Similar to the dual-layer filtering strategies proposed for correlated network systems by Zhao et al. [25], the ensemble and cross-validation framework adopted in this study effectively addresses the challenges of temporal and physiological correlations within multimodal datasets, ensuring reliable model evaluation [25].

### 2.10. Measures of Accuracy

To assess the models’ performance, a confusion matrix was created for each classification task. The rows denote the anticipated cases generated by the models. The columns denote the actual values. The definition of each numeral in the confusion matrix is as follows:

True Positives (TP): Instances identified by the model as lame cows that are indeed afflicted cases.

False Positives (FP): Instances identified by the model as lame cows that are, in fact, non-lame.

True Negatives (TN): Instances identified by the model as non-lame cows that are indeed non-lame.

False Negatives (FN): Instances identified by the model as non-lame cows that are, in fact, lame cases.

The subsequent metrics were derived from the confusion matrix:

Accuracy is defined as the ratio of correctly classified data to the total data by the model. Nonetheless, when the data are uneven, the outcomes may be excessively optimistic [26].

Accuracy=(TP+TN)/(TP+FP+FN+TN)

Sensitivity, also known as the True Positive Rate or recall measure, is the percentage of ill animals that the model accurately identifies.

Sensitivity=TP/(TP+FN)

Specificity: This is the proportion of negative cases that the model accurately identifies.

Specificity=TN/(FP+TN)

ROC–AUC: the ROC curve is a probabilistic graph that provides a thorough evaluation of the models’ efficacy. Probabilities are utilised in classification tasks to enable the categorisation of data at a defined threshold. The value was determined to be 0.5 in our study. This indicates that cows were categorised as lame cases when the chance above 0.5. Cows were categorised as non-lame if the probability was below 0.5. The false positive rate (FPR) is denoted by 1-Specificity on the x-axis of the ROC curve graph. Sensitivity is depicted on the vertical axis. The area beneath the ROC curve, ranging from 0 to 1, is termed the ROC–AUC. The ROC–AUC quantifies the model’s capacity to distinguish between positive and negative instances. The model’s capacity to differentiate between ill and non-ill instances is improved by an elevated ROC–AUC value.

Positive predictive value (PPV): This denotes the probability that the model accurately detects a cow as lame, notwithstanding the cow’s real illness. The prevalence of the condition within the sample affects it.

PPV=TP/(TP+FP)

Negative predictive value (NPV): This is the probability that the model erroneously classifies a cow as non-lame when it is, in fact, lame. This value is affected by the disease’s prevalence in the sample, akin to PPV.

NPV=TN/(TN+FN)

The MCC is an alternative statistic that, as seen by Chicco and Jurman [26], is not influenced by imbalanced datasets. The author described it as the calculus of the Pearson product-moment correlation coefficient between the actual and expected data. The scope of this metric is [−1, +1]. A MCC score nearing −1 signifies that the model generates exceptionally accurate predictions. An MCC nearing −1 signifies inadequate model performance. An MCC of 0 signifies performance equivalent to random prediction.

MCC=(TP×TN−FP×FN)/[(TP+FP)(TP+FN)(TN+FP)(TN+FN)]1/2 

Chicco and Jurman [26] suggested that the MCC value can be represented within the interval [0, 1]. This is known as normalised MCC (nMCC), computed using the formula nMCC = (MCC + 1)/2, where 0 signifies the worst-case scenario and 1 indicates the best-case scenario.

### 2.11. Feature Importance Analysis

Feature importance was assessed using two complementary approaches applied to the RF and Ensemble models: Gini importance (mean decrease in impurity) and permutation importance.

The Gini index was used to quantify the average decrease in node impurity contributed by each feature across all trees in the model, providing a measure of how strongly each variable contributed to model splitting decisions.

Permutation importance was calculated by randomly shuffling each feature and measuring the resulting decrease in model performance (ROC-AUC). This method provides a model-agnostic assessment of each variable’s contribution and helps mitigate potential biases associated with Gini-based rankings (e.g., preference for variables with more categories or higher variance).

The aim was to identify which physiological, behavioural, biochemical, and milk traits most strongly contributed to the model’s ability to discriminate between lame and healthy cows, thereby providing both predictive insight and biological interpretation.

Descriptive statistics were calculated using SPSS version 29.0 (IBM Corp., Armonk, NY, USA). Data normality was evaluated, and differences between lame and healthy cows were assessed using independent Student’s *t*-tests for normally distributed variables and one-way ANOVA where appropriate. For non-normally distributed variables, non-parametric tests (Mann–Whitney U) were applied. A 95% confidence interval (CI) was applied, and results were considered statistically significant when *p* < 0.05. Data are presented as means ± standard deviations for normally distributed variables, and as medians with interquartile ranges for non-normally distributed variables.

## 3. Results

This section provides a detailed comparison of six ML algorithms applied to the early identification of lameness in dairy cows. Key performance metrics—including accuracy, sensitivity, specificity, predictive values, and overall classification ability—are presented for each model. Moreover, the analysis highlights the most influential physiological, behavioural, blood-based, and milk quality indicators contributing to the classification accuracy, offering insights into their predictive importance for detecting lameness in early lactation.

### 3.1. Classification Model Performance Based on Normalised MCC

Table 4 presents the performance of six ML models in classifying lame and healthy dairy cows during early lactation, as evaluated by the nMCC. The nMCC metric is particularly well-suited for imbalanced datasets and offers a robust measure of overall model performance by considering true and false positives and negatives. Among the evaluated models, RF achieved the highest nMCC score of 0.94, indicating near-perfect predictive capability and strong agreement between predicted and actual classifications. Both the NN and the Ensemble model also exhibited excellent performance, each attaining an nMCC of 0.90, suggesting their high reliability in capturing complex nonlinear patterns associated with lameness. KNN algorithm followed closely with an nMCC of 0.78, demonstrating relatively strong discriminative ability. In contrast, SVM achieved a moderate nMCC of 0.71, indicating somewhat lower classification precision compared to the top-performing models. LR model, with an nMCC of 0.30, exhibited the weakest performance, likely due to its linear nature and limited capacity to model complex interactions among physiological, behavioural, and biochemical indicators. These findings underscore the superiority of tree-based and deep learning approaches for early detection of lameness in dairy cows, particularly when working with heterogeneous and multidimensional sensor-derived data.

### 3.2. Comparative Analysis of Classification Models and Influential Diagnostic Features

Table 5 presents a comprehensive evaluation of six ML models—RF, SVM, LR, NN, KNN, and an Ensemble model—for classifying dairy cows with and without lameness during early lactation. Among all tested models, RF achieved the highest performance, with a sensitivity of 92.73 ± 12.06 and a specificity of 100.00 ± 0.00, indicating excellent capability to correctly classify both healthy and lame cows. Its overall accuracy was 97.04 ± 4.91, and its area under the curve (AUC) (99.77 ± 0.68) and MCC (0.94 ± 0.10) confirmed its strong generalizability and robust predictive power. The NN model showed comparably high performance, with a sensitivity of 94.55 ± 11.64, specificity of 95.07 ± 6.04, and an AUC of 97.73 ± 5.73, suggesting its suitability for capturing complex nonlinear patterns related to lameness. The Ensemble model, which integrates predictions from RF, SVM, and NN, also achieved high scores across metrics, including an MCC of 0.90 ± 0.09 and AUC of 96.82 ± 4.43, confirming the benefits of model aggregation.

In contrast, LR demonstrated the weakest performance, with a sensitivity of 52.73 ± 9.79, specificity of 75.85 ± 8.75, and an AUC of only 74.89 ± 6.16, indicating limited utility in this complex classification task. SVM provided intermediate results, showing strong specificity (94.49 ± 5.09) but lower sensitivity (73.64 ± 11.10), and an AUC of 94.31 ± 5.89, reflecting a tendency to favour healthy classifications. KNN achieved moderate performance across all metrics, with a sensitivity of 89.09 ± 12.06, specificity of 88.38 ± 8.29, and an AUC of 89.39 ± 7.53.

**Figure 1:**
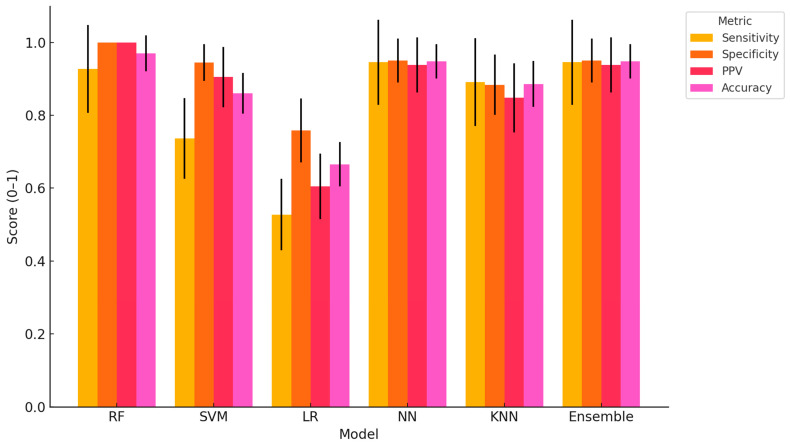
Performance metrics (Mean ± SD) of ML models for lameness detection in dairy cows. Grouped bar chart displaying sensitivity, specificity, positive predictive value (PPV), and accuracy (mean ± SD) of six classification models: Random Forest (RF), Support Vector Machine (SVM), Logistic Regression (LR), Neural Network (NN), k-Nearest Neighbors (KNN), and an Ensemble model. Metrics were derived using Monte Carlo cross-validation based on a dataset including physiological, behavioural, blood biomarker, and milk quality traits. RF, NN, and Ensemble models showed superior and stable classification performance.

Among the tested models, RF demonstrated the most consistent and outstanding performance, achieving near-perfect scores in all four metrics (sensitivity = 92.73 ± 12.06; specificity = 100.00 ± 0.00; PPV = 100.00 ± 0.00; accuracy = 97.04 ± 4.91) (Figure 1). NN and the Ensemble model also performed strongly, with almost identical metric profiles (e.g., accuracy = 94.84 ± 4.73; PPV = 93.85 ± 7.54), reflecting their robust ability to capture complex patterns in high-dimensional physiological, behavioural, and milk-based features. SVM displayed strong specificity (94.49 ± 5.09) and PPV (90.53 ± 8.28), but a lower sensitivity (73.64 ± 11.10), suggesting a tendency to under-detect lame cows. LR showed the weakest performance overall, particularly in sensitivity (52.73 ± 9.79) and PPV (60.46 ± 9.01), indicating a limited ability to generalize beyond linear patterns. KNN achieved intermediate results with balanced though slightly lower metrics compared to NN and the Ensemble model.

**Figure 2:**
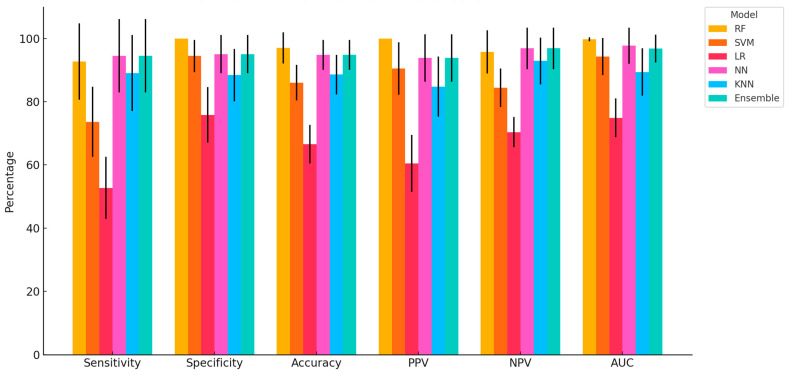
Comparative performance of six ML models in classifying lameness in dairy cows (Mean ± SD). Bar plot comparing the performance of Random Forest (RF), Support Vector Machine (SVM), Logistic Regression (LR), Neural Network (NN), k-Nearest Neighbors (KNN), and Ensemble models across six classification metrics: sensitivity, specificity, accuracy, positive predictive value (PPV), negative predictive value (NPV), and area under the curve (AUC). Values represent mean percentage scores ± standard deviation from repeated Monte Carlo cross-validations using physiological, behavioural, blood biomarker, and milk quality features for early-lactation lameness detection.

Among all models, RF consistently achieved the highest scores across most metrics, including perfect specificity and PPV (100.00 ± 0.00), high accuracy (97.04 ± 4.91), and AUC (99.77 ± 0.68), confirming its superior ability to correctly classify both lame and healthy cows with minimal error (Figure 2). NN and the Ensemble model closely followed, showing strong performance across all criteria, particularly with balanced sensitivity (94.55 ± 11.64), NPV (96.89 ± 6.53), and AUC values above 96%. SVM demonstrated good specificity and PPV but was notably lower in sensitivity (73.64 ± 11.10), suggesting its limited ability to detect all lame cows. KNN also performed relatively well, especially in accuracy and AUC, outperforming SVM in sensitivity (89.09 ± 12.06) but slightly underperforming in specificity. Conversely, LR consistently exhibited the lowest metrics among all models, particularly in sensitivity (52.73 ± 9.79) and AUC (74.89 ± 6.16), indicating poor discrimination capacity for the lameness condition based on complex, nonlinear physiological and biomarker signals.

This comparative visualization clearly illustrates that ensemble-based and nonlinear models (RF, NN, Ensemble) are more reliable and accurate in detecting early-lactation lameness than linear models, which are less adaptable to the multimodal and noisy nature of sensor and biological data.

### 3.3. Feature Importance Evaluation

To explore which parameters contributed most to the model’s decision-making, a feature importance analysis was performed using the Random Forest classifier. Variable importance was estimated through permutation analysis on the validation set, quantifying the decrease in predictive performance when each feature was randomly permuted. Although the analysis identified consistent trends, the overall ranking stability was affected by the moderate class imbalance (162 healthy vs. 110 lame cows) and by intercorrelations among several predictors—particularly milk composition traits (fat, protein, lactose) and biochemical parameters (GGT, AST, NEFA). As a result, importance scores exhibited small absolute differences, making precise ordering between features less meaningful. Future studies using larger datasets and model-agnostic interpretability techniques (e.g., SHAP analysis) could provide more stable feature ranking across models.

### 3.4. Formatting of Mathematical Components

Descriptive statistical analysis was conducted to compare a broad set of indicators between dairy cows diagnosed with lameness and clinically healthy counterparts during early lactation (Table 6).

Among environmental parameters, the THI and heat index showed no significant differences between groups (*p* = 0.722 and *p* = 0.686, respectively), indicating that lameness was not confounded by ambient climatic conditions in this dataset. However, significant physiological and behavioural deviations were observed. Water intake was significantly lower in lame cows (127.35 ± 29.77 L/day) compared to healthy cows (145.06 ± 29.92 L/day, *p* < 0.001), reflecting altered hydration or feeding behaviour possibly due to discomfort or reduced mobility. Reticulorumen temperature without drinking cycles and normal body temperature were slightly elevated in lame cows (*p* = 0.003 and *p* < 0.001, respectively), potentially indicating low-grade inflammation or altered thermoregulation. In contrast, overall cow activity and rumination time did not differ significantly between groups (*p* = 0.534 and *p* = 0.461), suggesting that compensatory behavioural adaptations may mask clinical signs in some cases.

Biochemically, lame cows exhibited significantly lower levels of AST, GGT, and LDH, with respective *p*-values of 0.001, <0.001, and <0.001. These findings may indicate hepatic strain or systemic inflammatory responses in association with lameness. Notably, NEFA concentrations were significantly elevated in lame cows (0.14 ± 0.14 mmol/L vs. 0.09 ± 0.08 mmol/L in healthy cows, *p* < 0.001), suggesting increased lipomobilization and metabolic stress. While TP and TRIG levels did not differ significantly between groups, Fe levels showed a borderline reduction in lame cows (*p* = 0.076).

Milk production traits revealed further disparities. Although milk yield was slightly higher in lame cows (37.68 ± 9.69 kg/day vs. 36.44 ± 8.88 kg/day), the difference was not statistically significant (*p* = 0.278), possibly due to large variability within groups. However, milk protein content was significantly lower in lame cows (3.21 ± 0.38%) compared to healthy cows (3.35 ± 0.27%, *p* < 0.001), while milk lactose was also lower in the lame group (4.71 ± 0.51% vs. 4.84 ± 0.18%, *p* = 0.003). No significant differences were detected for milk fat content, fat-to-protein ratio, or milk temperature.

## 4. Discussion

### 4.1. Main Features Associated with Clinical Lameness in Dairy Cattle

This study provides compelling evidence that ML algorithms, particularly RF, NN, and Ensemble models, can accurately classify dairy cows with clinically diagnosed lameness using a comprehensive, multimodal dataset encompassing physiological, behavioural, blood-based, and milk composition variables. The high classification accuracy achieved by RF (97.04%), coupled with perfect specificity and PPV, demonstrates the strong predictive capability of tree-based models in handling heterogeneous, nonlinear data. NN and the Ensemble model also performed consistently well across all performance metrics, further validating their capacity to capture complex biological interactions associated with lameness. Similar findings have been reported in other contexts; for example, in a study evaluating classification models for lameness treatment events, RF was compared with LR and Gaussian Naïve Bayes. Although the RF model achieved a more modest performance (AUC = 0.71) for lameness detection based on sensor-derived variables such as pedometer activity and feed intake, it was still recommended as a benchmark model owing to its interpretability and consistent predictive ability. Interestingly, other authors also observed that oversampling techniques did not enhance AUC, reinforcing RF’s robustness under different modelling conditions [27]. Indeed, previous research in dairy cattle health monitoring confirms the suitability of RF in such contexts: for example, Dineva et al. [28] applied RF to classify cow health status using heterogeneous IoT and sensor data and achieved an accuracy of 0.959, with recall 0.954 and precision 0.97 [28].

The superiority of RF and NN observed in this study aligns with previous research indicating that models capable of handling nonlinear, high-dimensional data outperform simpler linear methods in animal health applications [9,29,30]. In contrast, LR, a linear model, showed limited predictive value, with the lowest sensitivity (52.73%) and AUC (74.89%) among all tested models. This aligns with earlier findings, where logistic regression models for lameness detection generally achieved only moderate accuracy, with AUC values ranging from 0.70 to 0.77 [31]. One explanation for this limited performance is that LR assumes linear relationships between predictors and outcomes, whereas lameness in dairy cows arises from multi-factorial and nonlinear physiological processes. Pain-induced alterations in gait, weight redistribution, and locomotor asymmetry interact with changes in feed intake, rumination time, and milk yield, all of which are influenced by metabolic and inflammatory states [32]. Such complex interactions are not easily captured by linear models.

Among the models evaluated, SVM and KNN demonstrated intermediate performance. SVM showed high specificity (94.49%) but lower sensitivity (73.64%), suggesting a bias toward correctly identifying healthy animals while under-detecting truly lame cows. This could lead to an increased risk of false negatives in practical settings. KNN displayed balanced but slightly lower overall performance, which may be attributed to its sensitivity to feature scaling and data noise. In line with these observations, previous work has shown that an Long Short-Term Memory (LSTM) model trained on step-size feature vectors achieved a lameness detection accuracy of 98.57%, outperforming SVM, KNN, and Decision Tree Classifier (DTC) by margins of 2.93%, 3.88%, and 9.25%, respectively [33], further emphasizing the advantage of advanced nonlinear models in capturing gait-related abnormalities. Complementing our findings, Neupane et al. [34] evaluated ML models using accelerometer-derived behavioural data (day-to-day lying time, step counts, and their trends) for detecting lameness and the need for corrective or therapeutic claw treatments. They found that the ROCKET time-series classifier, particularly when combining conventional and slope features, significantly outperformed RF, Naïve Bayes, and LR—achieving accuracies > 90%, ROC–AUCs > 0.74, and F1-scores > 0.61 for identifying cows needing intervention [34]. Post et al. [27] developed classification models for both mastitis and lameness treatment events using daily individual sensor data—such as milking parameters, pedometer activity, feed and water intake, and body weight. They compared various ML methods (LR, SVM, KNN, Gaussian Naïve Bayes, Extra Trees (ET), and RF, and found the ET classifier achieved the highest mean AUC of 0.79 for mastitis and 0.71 for lameness, closely followed by Gaussian Naïve Bayes, LR, and RF—highlighting good interpretability alongside competitive performance [27]. A recent study by Lemmens et al. [35] demonstrated that integrating sensor data, automated milking system (AMS) parameters, and farm-level information substantially improved the detection of mild lameness (locomotion score ≥ 2). Their RF model achieved an accuracy of approximately 0.75, with a sensitivity of 0.72 and a specificity of 0.78. Importantly, eating time, low activity, medium activity, and activity trends differed significantly between lame and non-lame cows, whereas rumination time remained largely unaffected [35].

The Ensemble model, integrating predictions from RF, NN, and SVM, provided robust performance across all metrics and may serve as a practical compromise when aiming to balance specificity, sensitivity, and interpretability. Its consistent classification accuracy and relatively low variation across Monte Carlo cross-validations point to its potential as a stable and generalizable tool for on-farm decision support. Similarly, Yuhao Shen et al. [36] described an ensemble learning approach for detecting cow lameness, where an improved YOLOv8-Pose model was used to identify key points on the hooves, knees, hips, and head, and motion features were fused through a stacking ensemble method, achieving an overall accuracy of 97.2% [36].

The clinical relevance of the models’ performance is underscored by the biological interpretation of key input features. For example, lame cows exhibited significantly lower water intake and higher reticulorumen and body temperatures—traits likely reflective of discomfort, inflammatory responses, or altered metabolic function. Biomarkers such as NEFA, GGT, AST, and LDH were also significantly altered in lame cows, supporting the hypothesis that lameness is accompanied by systemic physiological and metabolic changes. Interestingly, Meléndez et al. [37] demonstrated that acute health disorders in dairy cows can induce measurable shifts in serum metabolic profiles, including GGT activity, thereby supporting the notion that alterations in this enzyme are part of a broader systemic response to disease rather than isolated anomalies [37]. Reduced AST levels observed in our study may also point toward compromised metabolic processes; however, it is important to acknowledge that the relationship between lameness and AST activity remains inconsistent in the literature, with some report describing unchanged values [38,39]. These findings concur with Praxitelous et al. [40], who documented higher GGT concentrations in lame cows during the puerperium period (25.83 vs. 23.56, *p* = 0.02) [40], as well as with un-targeted metabolomics work by He et al. [41], which identified lipid-metabolism metabolites as discriminative markers in lame dairy cattle [41]. Furthermore, scoping reviews such as Sadiq et al. [42] consistently list NEFA and liver enzymes among the most frequently studied biomarkers linked to lameness in dairy cows [42]. In addition, Dineva et al. [28] demonstrated that a RF classifier using heterogeneous sensor and physiological data achieved high predictive accuracy (0.959), recall (0.954) and precision (0.97)—evidence for the utility of combining diverse biological signals in health monitoring [28]. These discrepancies suggest that biochemical responses to lameness are likely influenced by the severity, chronicity, and underlying causes of locomotor impairment, as well as by concurrent metabolic and inflammatory conditions. Notably, elevated NEFA levels in lame cows suggest increased lipomobilization, which may reflect an underlying energy imbalance or stress-induced metabolic shift. These findings are consistent with prior studies that have identified NEFA and liver enzymes as important indicators of systemic inflammation and metabolic stress in dairy cattle.

From a milk production perspective, lame cows showed reduced milk protein and lactose content, further highlighting the systemic impact of lameness on metabolic efficiency and mammary gland function. Although overall milk yield was not significantly different, the compositional shifts in milk underline the potential of milk traits as non-invasive biomarkers for health monitoring. Kass et al. [43], in a study of Estonian Holstein cows, demonstrated that lame individuals produced significantly less milk overall, with concomitant decreases in milk protein and fat yield compared to their non-lame counterparts [43]. Furthermore, our previous studies showed that milk lactose dynamics are also altered around the onset of lameness: healthy cows had significantly lower lactose levels com-pared to lame cows both on the day of diagnosis (−2.15%) and seven days thereafter (−1.73%), suggesting that lameness is associated with transient changes in lactose synthesis and secretion [44]. Indeed, in the study by Jukna et al. [45], severe lameness was associated with a decrease in milk lactose concentration by 0.16 percentage points (*p* < 0.001) as lameness severity intensified, supporting the notion that lameness alters milk composition [45]. Moreover, Bonfatti et al. [46] explored the use of milk mid-infrared spectra to predict lameness scores, suggesting that deviations in milk spectral traits may reflect underlying metabolic disturbances, which in turn influence milk composition [46].

The effectiveness of our predictive models for lameness is underscored by the biological and physiological significance of their key input features. We observed that lame cows exhibit reduced water intake and elevated body temperature. The elevated body temperature is a clear physiological marker of inflammation, a process where the cow redirects energy and resources away from productive behaviours like milk synthesis and toward pain management and tissue repair. Further supporting this link, a separate study by Antanaitis et al. [47] demonstrated that changes in reticulorumen temperature patterns coincided with the onset of clinical lameness, highlighting the strong connection between these physiological indicators and the manifestation of the disease [47]. Therefore, our models’ reliance on these specific behavioural and physiological features is biologically justified, reinforcing their clinical relevance for early lameness detection.

Interestingly, rumination time and cow activity did not differ significantly between groups, which may suggest behavioural compensation in lame animals or highlight the difficulty of relying on these measures in isolation for lameness detection. Weigele et al. [48] observed that moderate lameness had no significant impact on rumination time, number of ruminating chews, or boluses, suggesting that basic rumination behaviour remains relatively stable despite locomotor impairment [48]. Likewise, Thorup et al. [49] reported that lameness did not affect daily rumination time, number of rumination events, or overall rumination behaviour, even though feeding behaviour was clearly altered. From a physiological standpoint, this stability may be explained by the cow’s strong homeostatic drive to maintain rumen function and fiber digestion, which are essential for sustaining microbial fermentation and volatile fatty acid production, even when mobility is compromised [50]. Consequently, lame cows may preserve rumination behaviour while reducing other energy-demanding activities, such as locomotion or the frequency of visits to the feed bunk [51]. These findings underscore that rumination and activity data, when used in isolation, may fail to capture the multifactorial nature of lameness, highlighting the need to integrate them with gait-related metrics, weight distribution patterns, or biochemical indicators to achieve more reliable detection. This further justifies the use of multimodal data integration in model development. The inclusion of both objective sensor data and clinically relevant biomarkers strengthens the interpretability and real-world applicability of the resulting models.

### 4.2. Reflections on Strengths, Limitations, and Scientific Outlook

One of the main strengths of this study lies in its integrative approach to lameness classification, combining physiological, behavioural, blood biochemical, and milk composition data into a comprehensive ML framework. This multimodal design reflects the multifactorial nature of lameness and allows for more biologically meaningful classification models than approaches that rely on single data streams. By evaluating six different algorithms and incorporating robust cross-validation, the study offers a clear and comparative picture of model performance and stability. The consistent superiority of Random Forest, Neural Networks, and the Ensemble model demonstrates the power of non-linear and ensemble methods to capture complex patterns in real-world, farm-derived data. Although preliminary feature importance analysis was conducted, the relatively limited sample size and intercorrelated structure of the dataset may have affected the stability of variable ranking. Future studies with larger, longitudinal datasets are needed to derive more robust interpretability measures.

Another important strength is the biological interpretability of the input features. Several of the indicators that differed significantly between lame and healthy cows—such as water intake, NEFA levels, liver enzymes (GGT, AST, LDH), milk protein, and lactose content—are not only statistically significant but also physiologically relevant, reinforcing the clinical validity of the model outputs. These findings support the growing role of sensor and biomarker-based monitoring systems in veterinary diagnostics.

Despite these contributions, the study has several limitations. The models do not predict the onset of lameness but rather classify cows as lame or healthy based on their current biological status. Nevertheless, it is essential to acknowledge that the models developed in this study serve as diagnostic aids rather than predictive tools. Because the data were collected at the time of clinical diagnosis, the models classify cows according to their current physiological and biochemical status and do not predict the future onset of lameness. This distinction is important for setting realistic expectations about their practical application. Moreover, the cross-sectional nature of the study limits conclusions about the temporal progression of lameness or the dynamics of the involved indicators over time.

Another constraint is the absence of external validation. While the models performed well under internal cross-validation, their generalizability to other farms, management systems, or cow populations remains to be tested. Additionally, behavioural variables such as rumination time and activity did not show significant differences between groups, possibly due to adaptation or masking behaviours in lame cows. This suggests that sensor-based behavioural indicators alone may not be sufficiently sensitive or specific for detecting lameness without additional physiological or biochemical context. It should also be noted that a moderate class imbalance existed in the dataset (healthy cows = 162; lame cows = 110). Although stratified sampling and balanced evaluation metrics such as MCC and nMCC were used to mitigate its effects, linear models like Logistic Regression may still be more sensitive to such imbalance, which could have contributed to their comparatively lower performance.

Looking ahead, future research should focus on longitudinal data collection and repeated measurements across the disease trajectory to evaluate whether these multimodal ML models can be adapted for pre-clinical prediction, relapse monitoring, or progression assessment. External validation using independent datasets from commercial farms is also essential to assess model scalability and real-world applicability. Lastly, integrating genetic, environmental, and management variables could further refine model accuracy and resilience across diverse production settings.

In summary, this study contributes a robust, multimodal framework for clinical classification of lameness in dairy cows and highlights the utility of combining biosensor data with milk and blood biomarkers. While not predictive, the models offer a promising diagnostic support tool that can enhance the objectivity, consistency, and efficiency of veterinary decision-making within precision dairy systems.

## 5. Conclusions

This study confirms the effectiveness of ML models, particularly RF, NN, and Ensemble methods, in accurately classifying clinically lame dairy cows using multimodal data collected at the time of diagnosis. The RF model achieved the highest accuracy (97.04%) and perfect specificity and PPV, while NN and the Ensemble model also demonstrated strong and consistent diagnosis performance. In contrast, linear models such as LR showed substantially lower sensitivity and diagnostic value. Significant differences in physiological, biochemical, and milk-related indicators between lame and healthy cows—including elevated NEFA concentrations, liver enzymes, and body temperature, together with reduced milk protein and lactose—confirm the biological relevance of the selected features. These findings highlight the multifactorial nature of lameness and justify the integration of such parameters into data-driven diagnostic tools.

While the models were trained on data collected during clinical lameness diagnosis—and therefore do not constitute pre-clinical prediction—they provide a robust, objective, and high-accuracy diagnostic framework for detecting lameness. This approach represents a significant step forward in precision dairy health monitoring and establishes a foundation for future integration of multimodal biosensor and biomarker data into intelligent herd management systems.

## Figures and Tables

**Table 1:** Chemical composition of TMR for lactating dairy cows.

TMR Component	Value
Dry matter	50.7%
Neutral detergent fiber	28.3% of DM
Acid detergent fiber	19.8% of DM
Net lactation energy	1.6 Mcal/kg
Crude protein	15.8% of DM
Non-fiber carbohydrates	38.7% of DM

TMR—total mixed ratio, DM—dry matter.

**Table 2:** Composition of TMR for lactating dairy cows.

TMR Component	Value
Mineral mix	6.0%
Grain concentrate mash	49.0%
Grass silage	10.0%
Corn silage	31.0%
Alfalfa grass hay	4.0%

TMR—total mixed ratio.

**Table 3:** Summary of collected variables and laboratory analyses.

Type of Measurement	Measured Variable	Unit	Instrument
Production parameters	Milk yield	kg/day	DeLaval milking robots
Milk parameters	Milk composition (fat, protein, lactose, milk fat-to-protein ratio)	%	Brolis HerdLine in-line milk analyzer (Brolis Sensor Technology, Vilnius, Lithuania)
Environmental parameters	THI	-	SmaXtec climate station (SmaXtec Animal Care GmbH, Graz, Austria)
Behavioural parameters	Rumination time	min/day	SmaXtec bolus (SmaXtec Animal Care GmbH, Graz, Austria)
	Activity	h/day	SmaXtec bolus
	Water intake	L/day	SmaXtec bolus
Physiological parameters	Body temperature	°C	SmaXtec bolus
	Milk temperature	°C	BROLİS HerdLine in-line milk analyzer (Brolis Sensor Technology, Vilnius, Lithuania)
Blood biomarkers	AST	U/L	Randox clinical chemistry (RX Daytona, Randox Laboratories Ltd., London, UK)
	NEFA	mmol/L	Randox clinical chemistry
	GGT	U/L	Randox clinical chemistry
	LDH	U/L	Randox clinical chemistry
	TP	G/L	Randox clinical chemistry
	TRIG	mmol/L	Randox clinical chemistry
	Fe	µmol/L	Randox clinical chemistry

THI—temperature-humidity index; NEFA—nonesterified fatty acids; AST—aspartate aminotransferase activity; Fe—iron; GGT—gamma-glutamyl transferase activity; LDH—lactate dehydrogenase; TP—total protein; TRIG—triglycerides.

**Table 4:** Performance of classification models based on nMCC obtained through 10 Monte Carlo cross-validations for identifying lameness and healthy cows during early lactation.

Model ^2^	nMCC ^1^
RF	0.94
SVM	0.71
LR	0.30
NN	0.90
KNN	0.78
Ensemble	0.90

^1^ Values correspond to the normalised Matthews correlation coefficient (nMCC) obtained by the models in classifying cows with or without lameness based on physiological, behavioural, blood, and milk quality traits. The nMCC ranges from 0 (random prediction) to 1 (perfect classification) and provides a robust evaluation metric even for imbalanced data. ^2^ RF—random forest, SVM—support vector machine, NN—neural network, LR—logistic regression, KNN—k-nearest neighbors, Ensemble—integrated model combining predictions from RF, SVM, and NN.

**Table 5:** Performance metrics of ML models for lameness classification in dairy cows during early lactation.

Model	Sensitivity	Specificity	Accuracy	PPV	NPV	AUC	MCC
RF	92.73 ± 12.06	100.00 ± 0.00	97.04 ± 4.91	100.00 ± 0.00	95.78 ± 6.85	99.77 ± 0.68	0.94 ± 0.10
SVM	73.64 ± 11.10	94.49 ± 5.09	86.04 ± 5.64	90.53 ± 8.28	84.42 ± 6.06	94.31 ± 5.89	0.71 ± 0.12
LR	52.73 ± 9.79	75.85 ± 8.75	66.53 ± 6.09	60.46 ± 9.01	70.39 ± 4.79	74.89 ± 6.16	0.30 ± 0.13
NN	94.55 ± 11.64	95.07 ± 6.04	94.84 ± 4.73	93.85 ± 7.54	96.89 ± 6.53	97.73 ± 5.73	0.90 ± 0.09
KNN	89.09 ± 12.06	88.38 ± 8.29	88.61 ± 6.27	84.79 ± 9.52	92.91 ± 7.39	89.39 ± 7.53	0.78 ± 0.13
Ensemble	94.55 ± 11.64	95.07 ± 6.04	94.84 ± 4.73	93.85 ± 7.54	96.89 ± 6.53	96.82 ± 4.43	0.90 ± 0.09

Mean ± standard deviation of performance metrics for six classification models—Random Forest (RF), Support Vector Machine (SVM), Logistic Regression (LR), Neural Network (NN), k-Nearest Neighbors (KNN), and an Ensemble model—based on 10-fold Monte Carlo cross-validation. Metrics include Sensitivity, Specificity, Accuracy, Positive Predictive Value (PPV), Negative Predictive Value (NPV), Area Under the Curve (AUC), and Matthews Correlation Coefficient (MCC). Classification was based on physiological, behavioural, blood biomarker, and milk quality traits to distinguish lame cows (n = 1) from healthy cows (n = 0).

**Table 6:** Descriptive statistics of physiological, behavioral, and biochemical parameters in healthy and lame dairy cows during early lactation.

Descriptives										
Traits	Cow Group	N Records	Mean	Std. Deviation	Std. Error	95% Confidence Interval for Mean		Minimum	Maximum	Significant
						Lower Bound	Upper Bound			
THI	Healthy	162	61.05	3.17	0.25	60.56	61.54	56.69	64.53	0.722
	Lameness	110	61.19	3.26	0.31	60.57	61.81	56.69	64.53	
Water intake (L/day)	Healthy	162	145.06	29.92	2.35	140.42	149.70	74.93	225.72	<0.001
	Lameness	110	127.35	29.77	2.84	121.73	132.98	72.80	179.19	
Cow activity (h/day)	Healthy	162	7.32	3.36	0.26	6.80	7.84	2.31	20.01	0.534
	Lameness	110	7.06	3.31	0.32	6.43	7.69	1.31	15.92	
Body temperature (°C)	Healthy	162	39.43	0.13	0.01	39.41	39.45	39.14	39.72	<0.001
	Lameness	110	39.49	0.15	0.01	39.46	39.52	39.16	40.00	
Rumination time (min/day)	Healthy	162	493.93	66.39	5.22	483.63	504.23	327.62	610.13	0.461
	Lameness	110	488.14	59.08	5.63	476.97	499.30	329.43	575.43	
AST	Healthy	162	95.75	32.92	2.59	90.65	100.86	39.70	218.60	0.001
	Lameness	110	84.37	21.01	2.00	80.40	88.34	48.60	144.20	
Fe	Healthy	162	22.74	5.52	0.43	21.88	23.60	12.40	39.40	0.076
	Lameness	110	21.43	6.54	0.62	20.19	22.67	6.90	36.60	
GGT	Healthy	162	38.00	14.23	1.12	35.79	40.21	12.50	77.50	<0.001
	Lameness	110	32.19	8.90	0.85	30.51	33.87	15.90	49.90	
LDH	Healthy	162	1393.41	277.32	21.79	1350.38	1436.44	911.00	2471.00	0.001
	Lameness	110	1284.51	257.48	24.55	1235.85	1333.17	935.00	1967.00	
NEFA	Healthy	162	0.09	0.08	0.01	0.07	0.10	0.02	0.42	<0.001
	Lameness	110	0.14	0.14	0.01	0.11	0.16	0.02	0.58	
TP	Healthy	162	80.52	6.76	0.53	79.47	81.57	68.90	100.40	0.397
	Lameness	110	81.26	7.43	0.71	79.85	82.66	62.70	101.10	
TRIG	Healthy	162	0.09	0.02	0.00	0.08	0.09	0.00	0.14	0.294
	Lameness	110	0.09	0.03	0.00	0.09	0.10	0.05	0.15	
Milk yield (kg)	Healthy	162	36.44	8.88	0.70	35.06	37.82	15.73	56.57	0.278
	Lameness	110	37.68	9.69	0.92	35.85	39.51	20.40	60.00	
Milk temperature (°C)	Healthy	162	36.42	0.59	0.05	36.33	36.51	34.90	37.43	0.880
	Lameness	110	36.43	0.88	0.08	36.26	36.60	33.82	37.82	
Milk fat (%)	Healthy	162	4.03	0.52	0.04	3.95	4.11	2.78	5.18	0.148
	Lameness	110	3.91	0.83	0.08	3.75	4.06	1.77	6.88	
Milk protein (%)	Healthy	162	3.35	0.27	0.02	3.31	3.39	2.75	3.99	<0.001
	Lameness	110	3.21	0.38	0.04	3.14	3.28	1.51	3.84	
Milk fat-to-protein ratio	Healthy	162	1.20	0.14	0.01	1.18	1.22	0.80	1.55	0.431
	Lameness	110	1.22	0.23	0.02	1.18	1.26	0.62	1.96	
Milk lactose (%)	Healthy	162	4.84	0.18	0.01	4.82	4.87	4.43	5.19	0.003
	Lameness	110	4.71	0.51	0.05	4.62	4.81	2.17	5.08	

NEFA—nonesterified fatty acids; AST—aspartate aminotransferase activity; Fe—iron; GGT—gamma-glutamyl transferase activity; LDH—lactate dehydrogenase; TP—total protein; TRIG—triglycerides.

## Data Availability

All pertinent data are presented within the manuscript. The machine learning scripts developed for this study were written in Python and can be obtained from the corresponding author upon reasonable request to support transparency and reproducibility of the findings.

## Abbreviations

The following abbreviations are used in this manuscript:

ML	Machine learning
RF	Random Forest
SVM	Support Vector Machine
NN	Neural Network
KNN	K-nearest neighbors
LR	Logistic Regression
NEFA	Non-esterified fatty acids
AST	Aspartate aminotransferase
LDH	Lactate dehydrogenase
GGT	Gamma-glutamyl transferase
nMMC	Normalized Matthews correlation coefficient
BHB	Beta-hydroxybutyrate
PLF	Precision livestock farming
TRIG	Triglycerides
ROC	Receiver operating characteristic
AUC	Area under the curve
MCC	Matthews Correlation Coefficient
FP	False Positives
TN	True Negatives
FN	False Negatives
FPR	False Positive Rate
PPV	Positive predictive value
NPV	Negative predictive value
TP	Total protein
Fe	Iron
LSMU	Lithuanian University of Health Sciences
TMR	Total mixed ration
DM	Dry matter
THI	Temperature–humidity index
RMSEP	Root mean square error of prediction
RBF	Radial Basis Function
TP	True Positives
Cl	Confidence interval
LSTM	Long Short-Term Memory
DTC	Decision Tree Classifier
ET	Extra Trees
AMS	Automated milking system
